# Sarcome épithélioide pleural, à propos d'un cas avec revue de la littérature

**DOI:** 10.11604/pamj.2016.25.65.8267

**Published:** 2016-10-03

**Authors:** Hajar Ouahbi, Youssra Akasbi, Karima Oualla, Bouchra Amara, Achraf Chatar, Siham Tizniti, Hind Fatemi, Fatima Zahra Lemrabet, Samia Arifi, Nawfal Mellas

**Affiliations:** 1 Service d’Oncologie Médicale, CHU Hassan II, Université Sidi Mohammed Ben Abdellah, Fès, Maroc; 2 Service de Pneumologie, CHU Hassan II, Fès, Maroc; 3 Service de Radiologie, CHU Hassan II, Fès, Maroc; 4 Service d’Anatomie Pathologie, CHU Hassan II, Fès, Maroc

**Keywords:** Sarcome épithélioide, type proximal, anatomopathologie, chimiothérapie, Epithelioid sarcoma, proximal type, chemotherapy

## Abstract

Le sarcome épithélioide proximal d'origine pleural révélé par un épanchement pleural reste rare dans la littérature, nous rapportons le cas d’une jeune patiente, admise au service d’oncologie médicale pour prise en charge d’un sarcome épithélioide pleural proximal métastatique. A la fin du premier cycle de chimiothérapie, la patiente est décédée dans un tableau de détresse respiratoire aiguë. Notre observation illustre le caractère potentiellement trompeur et agressif du sarcome épithélioide présentant un piège clinique pouvant mettre en jeu le pronostic vital et dont le diagnostic positif est strictement anatomopathologique.

## Abstract (en)

Proximal epithelioid sarcoma (PES) originating from the pleura is a clinical entity rarely reported in the literature. We report the case of a young patient with immediately metastatic proximal epithelioid sarcoma (PES) treated at the Department of Medical Oncology, Fes. Treatment consisted of chemotherapy based on doxorubicin and ifosfamide. After the first cycle of chemotherapy, the disease led to a fatal outcome. Our case study highlights the potentially aggressive behaviour of PES which represents a clinical trap and can be life-threatening.

## Introduction

Le Sarcome épithélioide de type proximal représente moins de 1% de l’ensemble des sarcome des tissus mous, la localisation pleurale comme site primaire reste un cas exceptionnel rapportée dans la littérature. L’objectif de cet article est de présenter un cas rare d’un sarcome épithélioide pleural métastatique, tout en rapportant une revue générale sur l’épidémiologie, le diagnostic, le traitement et les facteurs pronostiques du sarcome épithélioide proximal.

## Patient et observation

**Figure 1:**
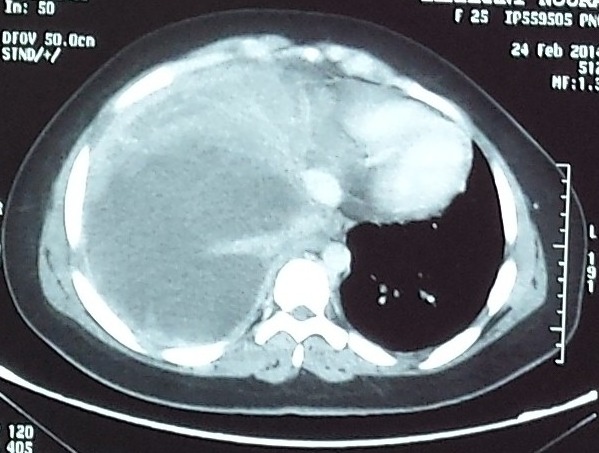
Epaississement pleural sans masse individualisable associé à un épanchement pleural liquidien cloisonné sur un scanner thoracique

**Figure 2:**
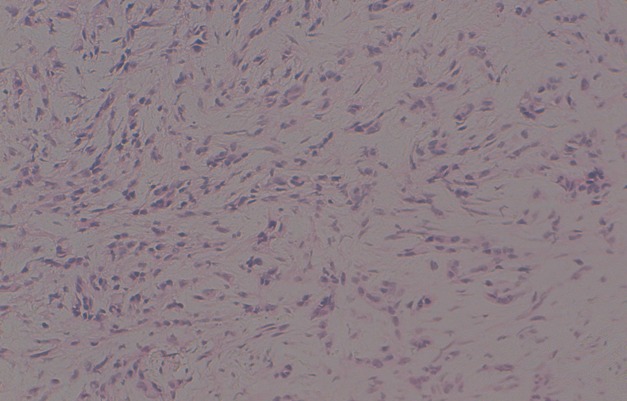
HES x 20: prolifération tumorale d’architecture diffuse. Les cellules sont allongées d’allure épithélioides, atypiques avec quelques figures de mitoses

**Figure 3:**
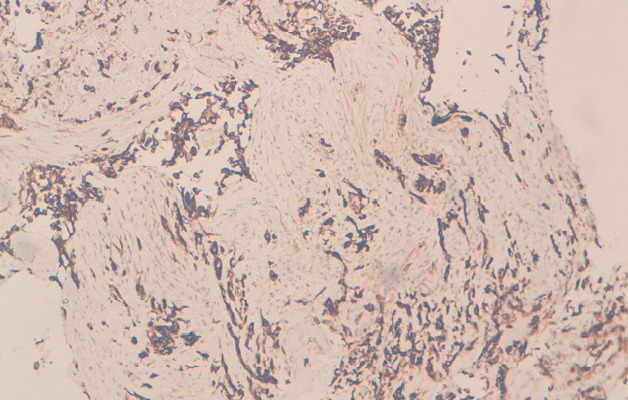
Marquage positif des cellules tumorales par l’'anticorps anti CD34

**Figure 4:**
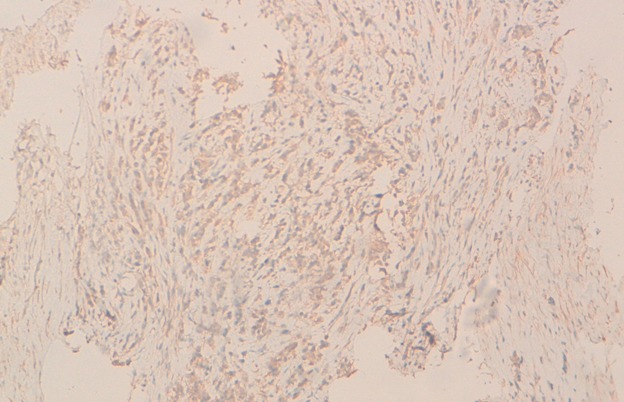
Marquage positif des cellules tumorales par l’'anticorps anti cytokératine 8/18

Il s'agit d'une patiente âgée de 25ans, ayant comme antécédents tabagisme passif depuis 10 ans, qui a présenté en Janvier 2014 une dyspnée d'aggravation progressive associé à des douleurs thoraciques type pleural. L'examen physique à l'admission a objectivé une patiente polypniéque à 30 cycles/minute, tachycarde à 138 battement par minute, SaO2 à 92% à l'aire ambiant, apyrétique, avec un syndrome d'épanchement liquidien de l'hémi thorax droit, associé à une douleur exquise à la palpation de la 3^ème^, 4^ème^ et la 5^ème^ cotes droites, sans autre anomalies associés; notamment un examen dermatologique qui était normale. Une radiographie thoracique était réalisée revenant en faveur d'une opacité de tonalité hydrique de l'hémi-champ thoracique droit, complété par une TDM(tomodensitométrie) thoracique objectivant un épanchement pleural liquidien de grande abondance cloisonné associé à un épaississement pleural sans masse individualisable (Figure 1). La thoracoscopie avec la réalisation d'une biopsie pleurale était en faveur d'un sarcome épithélioide type proximal figure 3, 4, dont l'immuno histochimie était en faveur d'une forte expression des anticorps anti-vimentine, anti CD34, anti-CK19 et faiblement les anticorps anti-CK7 et anti CK8 /18. Le marquage des cellules tumorales au CD99 était positif mais l'étude FISH était négatif, ainsi que la calrétinine, la CK 5 /6, les marqueurs lymphoïdes, les marqueurs neuroendocrine, les marqueurs mélaniques et la protéine S100 (Figure 2, Figure 3, Figure 4). Le bilan d'extension a montré des métastases ganglionnaires et pulmonaires. La décision thérapeutique prise en RCP (réunion de concertation pluridisciplinaire) était une chimiothérapie systémique à base de la bithérapie: doxorubicine 60mg/m^2^ à J1 et l'Ifosfamide à la dose de 3g/m^2^ sur 3 jours: J1=J21. L'évolution était marquée par le décès de la patiente quelques jours après la fin du premier cycle de sa chimiothérapie, dans un tableau de détresse respiratoire non jugulé par les mesures de réanimation.

## Discussion

Le Sarcome épithélioide représente 1% à 2% de l'ensemble des sarcome des tissus mous [1]. La première description de sa forme classique distale été faite par Enzinger en 1970, tandis que le type proximal ou type axial a été reconnu plus tard en 1997 [2]. Le diagnostic du sarcome épithélioide est exclusivement anatomopathologique, qui doit impérativement réalisé par un anatomopathologiste expérimenté en spécialité d'oncologie. Sur le plan histologique, les cellules de type proximal différent de la forme classique (distale) du sarcome épithélioïde par la présence de cellules tumorales de plus grande taille avec un cytoplasme plus abondant, des zones de nécrose, et un pléomorphisme nucléaire plus marqué, une proportion plus importante de cellules épithélioïdes montrant souvent un phénotype rhabdoïde, et l'absence habituelle d'architecture de type «granulome annulaire» [3]. L'ensemble de ces cellules tumorales montre un marquage positif pour la vimentine, la cytokératine et l'EMA (antigène épithélial membranaire). 50% des sarcomes épithélioïdes sont CD34 positifs, la Desmine peut être positive dans certains cas [4]. Le Diagnostic différentiel pour la forme proximale peut se poser avec un angiosarcome épithélioïde, un schwannome malin épithélioïde, un rhabdomyosarcome épithélioïde, une tumeur rhabdoïde, un mélanome et un carcinome indifférencié sans oublier le diagnostic différentiel avec une métastase d'un carcinome indifférencié [5]. Le traitement du sarcome épithélioide métastatique reste encore bien établie dans la littérature, la place de la chirurgie est réservé surtout pour les tumeurs résécables, il consiste à une résection chirurgicale large R0, tout en évitant les récidives locales qui sont très fréquentes [2, 5, 6]. La radiothérapie est rarement indiquée dans le type proximal métastatique, elle est surtout indiquée en cas de type distal, mais sans bénéfice en matière de survie globale [2]. L'indication et l'efficacité de la chimiothérapie dans le sarcome épithélioide métastatique restent insuffisamment prouvées [7]. Un nombre limité des essais cliniques et des études rétrospectives ont montré la résistance de ce type (proximal) au traitement systémique par rapport au type distal [6]. Les protocole de la chimiothérapie systémique ne sont pas bien codifiés, ainsi, la chimiothérapie systémique proposée, selon les écoles savantes, est celle délivrée au sarcome des tissus mou à différentiation incertaine ou en se référant aux essais cliniques des sarcomes du tissu mou incluant le sarcome épithélioide [7]. Les régimes de polychimiothérapie les plus couramment utilisés sont la doxorubicine et l'ifosfamide, plus au moins la dacarbazine [7]. Les bénéfices de thérapies ciblés tel que le Pazopanib en matière de SSP et SG étaient démontrés pour la première fois par l'étude de Palette qui a inclus le sarcome épithélioide dans les sous types des sarcomes des tissus mous métastatiques [8], ainsi, des essais cliniques pour identifier les caractéristiques moléculaires et les cibles thérapeutiques du sarcome épithélioide sont déjà publiés [9], ce qui encourage d'avoir à l'avenir des molécules plus spécifiques du sarcome épithélioide proximal métastatique. Sur le plan évolutif, ce sous type des sarcomes des tissus mou est plus agressif que la variante distale [3, 8, 10]. Le Pronostic est dépend surtout de la taille de la tumeur, la présence des caractéristiques rhabdoïde histologiquement et la réponse au traitement [2].

## Conclusion

Le sarcome épithélioide de type proximal métastatique est rarement rapporté dans la littérature dont la localisation pleurale et la présence de métastases pulmonaires sans masse tumorale évidente est exceptionnellement décrite, nous rapportons le premier cas dans la littérature, le diagnostic est obligatoirement anatomopathologique, le traitement est multidisciplinaire et palliatif, consiste surtout à améliorer la qualité de vie du malade, son pronostic est sombre, avec une agressivité importante qui peut mettre en jeu le pronostic vital, ce dernier peut être amélioré par une stratégie diagnostique et thérapeutique bien codifiés, y compris surtout une réunion de concertation pluridisciplinaire à chaque étape de la prise en charge.
